# Consensus statement on renal denervation by the Joint Committee of Japanese Society of Hypertension (JSH), Japanese Association of Cardiovascular Intervention and Therapeutics (CVIT), and the Japanese Circulation Society (JCS)

**DOI:** 10.1007/s12928-024-01017-1

**Published:** 2024-07-30

**Authors:** Kazuomi Kario, Hisashi Kai, Hiromi Rakugi, Satoshi Hoshide, Koichi Node, Yuichiro Maekawa, Hiroyuki Tsutsui, Yasushi Sakata, Jiro Aoki, Shinsuke Nanto, Hiroyoshi Yokoi

**Affiliations:** 1 Division of Cardiovascular Medicine, Jichi Medical University School of Medicine, 3311-1 Yakushiji, Shimotsuke, Tochigi, 329-0498 Japan; 2 Department of Cardiology, Kurume University Medical Center, Fukuoka, Japan; 3 Osaka Rosai Hospital, Sakai, Japan; 4 Osaka University, Suita, Japan; 5 Department of Cardiovascular Medicine, Saga University, Saga, Japan; 6 Division of Cardiology, Internal Medicine III, Hamamatsu University School of Medicine, Hamamatsu, Japan; 7 Department of Cardiovascular Medicine, Faculty of Medical Sciences, Kyushu University, Fukuoka, Japan; 8 Department of Cardiovascular Medicine, Osaka University Graduate School of Medicine, Suita, Osaka Japan; 9 Department of Cardiovascular Medicine, St. Luke’s International Hospital, Tokyo, Japan; 10 Department of Cardiovascular Medicine, Nishinomiya Municipal Central Hospital, Hyogo, Japan; 11 Cardiovascular Center, Fukuoka Sanno Hospital, Fukuoka, Japan

**Keywords:** Hypertension, Renal denervation, Resistant hypertension, Consensus statement

## Abstract

**Abstract:**

This is the first consensus statement of the Joint Committee on Renal Denervation of the Japanese Society of Hypertension (JSH)/Japanese Association of Cardiovascular Intervention and Therapeutics (CVIT)/Japanese Circulation Society (JCS). The consensus is that the indication for renal denervation (RDN) is resistant hypertension or “conditioned” uncontrolled hypertension, with high office and out-of-office blood pressure (BP) readings despite appropriate lifestyle modification and antihypertensive drug therapy. “Conditioned” uncontrolled hypertension is defined as having one of the following: 1) inability to up-titrate antihypertensive medication due to side effects, the presence of complications, or reduced quality of life. This includes patients who are intolerant of antihypertensive drugs; or 2) comorbidity at high cardiovascular risk due to increased sympathetic nerve activity, such as orthostatic hypertension, morning hypertension, nocturnal hypertension, or sleep apnea (unable to use continuous positive airway pressure), atrial fibrillation, ventricular arrythmia, or heart failure. RDN should be performed by the multidisciplinary Hypertension Renal Denervation Treatment (HRT) team, led by specialists in hypertension, cardiovascular intervention and cardiology, in specialized centers validated by JSH, CVIT, and JCS. The HRT team reviews lifestyle modifications and medication, and the patient profile, then determines the presence of an indication of RDN based on shared decision making with each patient. Once approval for real-world clinical use in Japan, however, the joint RDN committee will update the indication and treatment implementation guidance as appropriate (annually if necessary) based on future real-world evidence.

**Graphical Abstract:**

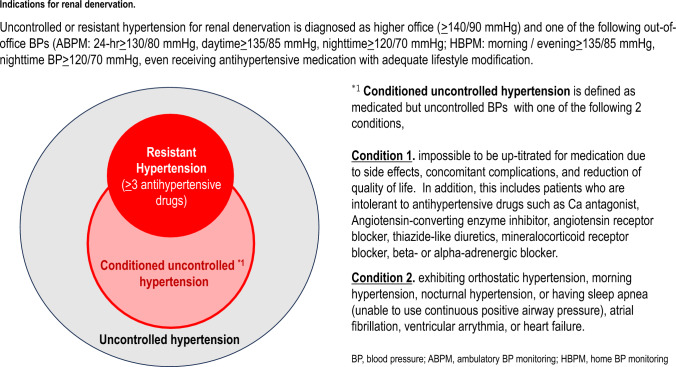

## Introduction

**Fig. 1:**
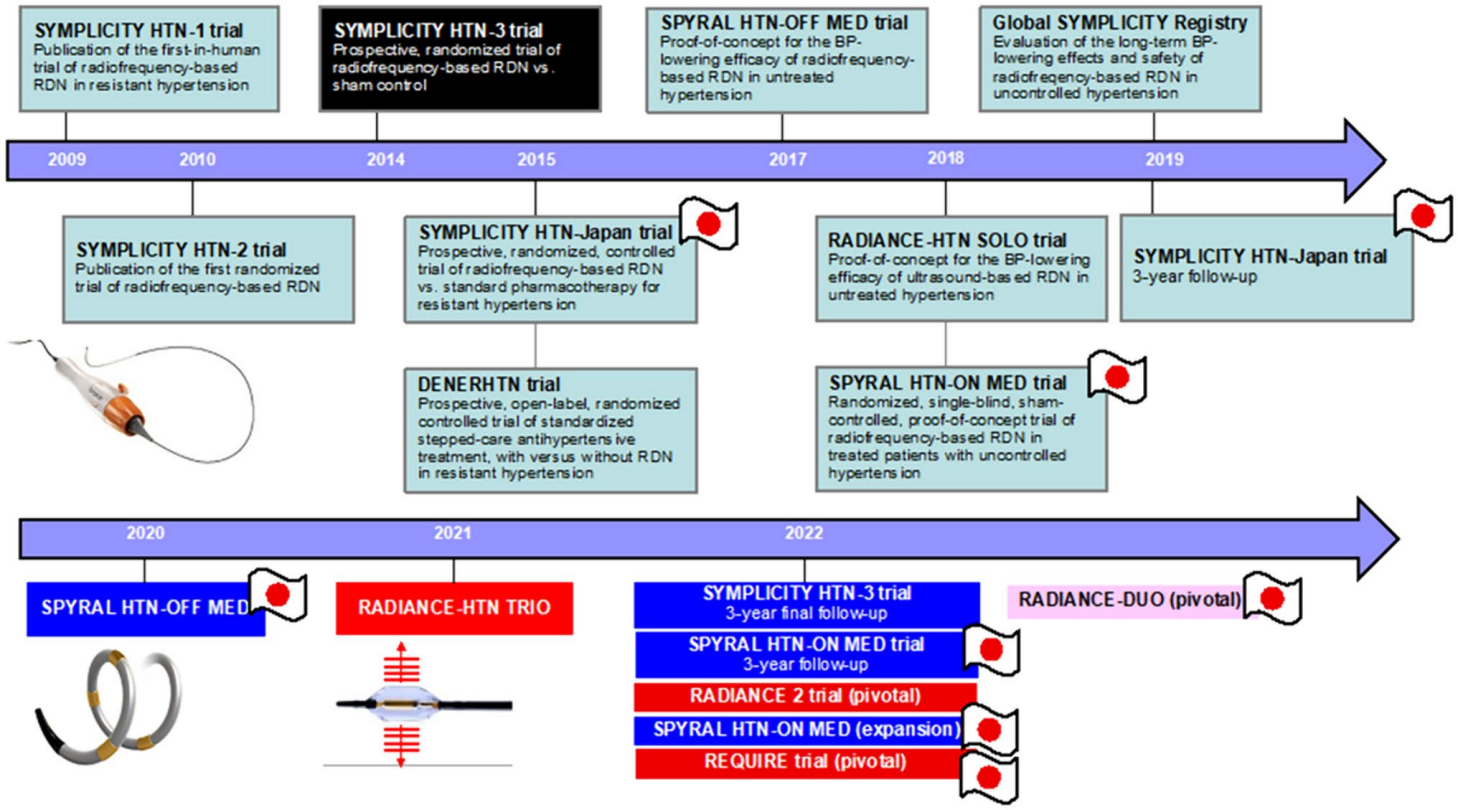
History of clinical trials investigating renal denervation (adapted and updated from Kario et al. Cardio Discov 2021;1:112–127)

Renal denervation (RDN) is an antihypertensive treatment that has a novel mechanism of action, acting on the central nervous system by denervating sympathetic afferent pathways [1–3]. The first “proof-of-principle” clinical trial of transcatheter RDN showed that treatment was associated with a marked reduction in blood pressure (BP) in patients with resistant hypertension [4]. While the results of the first pivotal trial of the first generation of radiofrequency-based RDN (SYMPLICITY HTN-2) were positive, this was an open-label trial with no sham control group [5]. Subsequently, the first sham-controlled trial of radiofrequency RDN, SYMPLICITY HTN-3, failed to document a significant difference in systolic BP (SBP) reduction between RDN and sham groups at 6 months after the procedure in patients with resistant hypertension [6]. Since then, there have been many other sham-controlled trials of both the second generation of radiofrequency- and ultrasound-based RDN in a variety of hypertensive patient populations (Fig. 1) [7–12]. Many of these have reported positive findings, with significantly greater reductions in BP in the RDN versus control group [7–9, 11]. Japan contributed to several of the key clinical trials, both with [8, 10, 12] and without [13, 14] a sham control group.

Based on the available data from the above trials, the US Food and Drug administration approved (FDA) approved both the SYMPLICITY SPYRAL radiofrequency RDN system and the PARADISE ultrasound RDN system for the adjunctive treatment of hypertension in patients with hypertension for whom lifestyle modifications and antihypertensive drug therapy do not adequately control BP.

The 2023 European Society of Hypertension (ESH) guidelines make a class II recommendation for the use of RDN in patients with uncontrolled hypertension [15], and consensus statements about RDN have been published by several societies and working groups [16–19]. This article details the Joint Consensus Statement on Renal Denervation Therapy in Japan 2024, developed by the Japanese Society of Hypertension (JSH), the Japanese Association of Cardiovascular Intervention and Therapeutics (CVIT), and the Japanese Circulation Society (JCS).

### Process and pre-procedure management

**Fig. 2:**
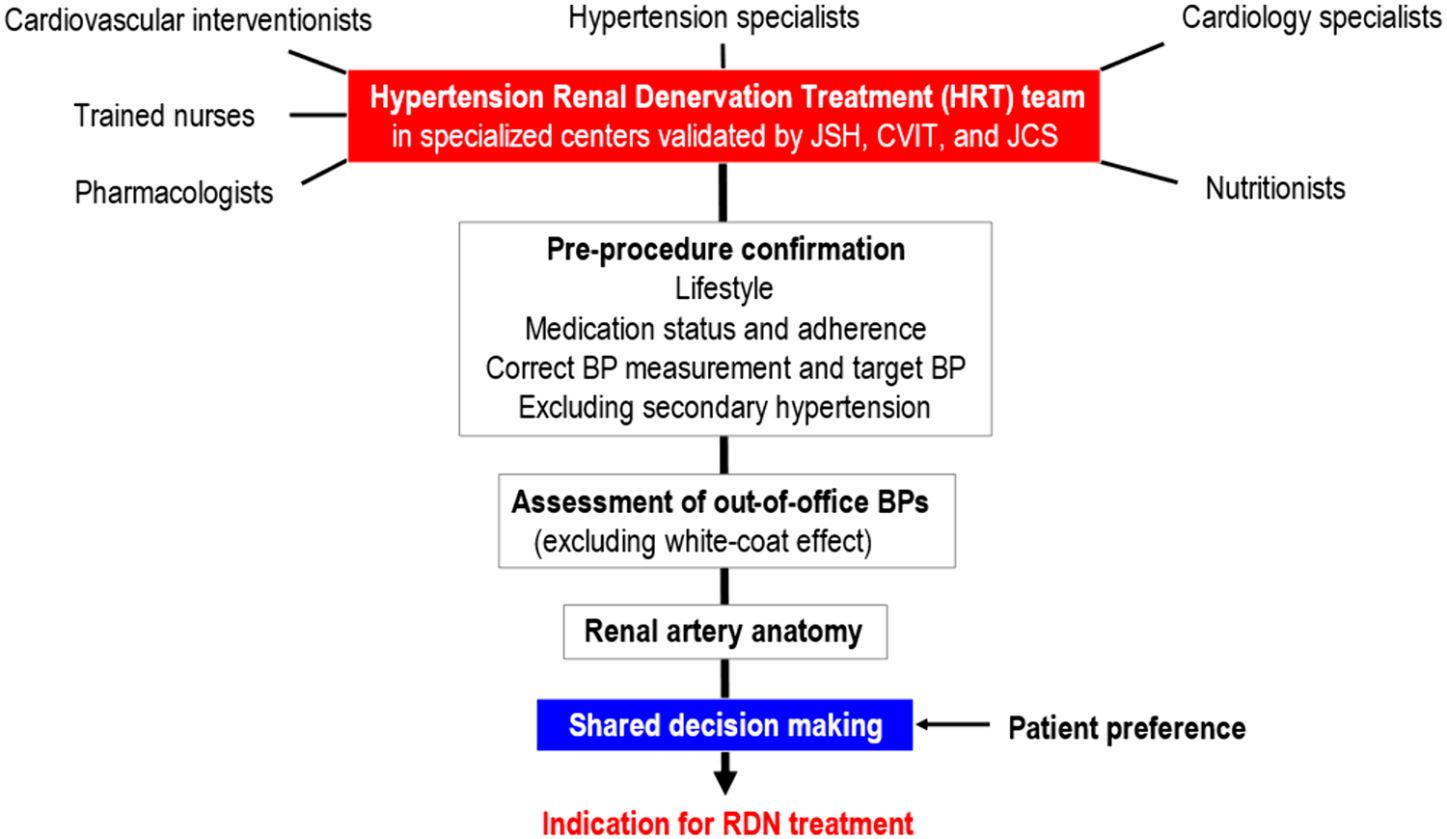
Process to determine the presence of an indication for renal denervation. *BP* blood pressure, *CVIT* Japanese Association of Cardiovascular Intervention and Therapeutics, *JCS* Japanese circulation society, *JSH* Japanese society of hypertension, *RDN* renal denervation

A multidisciplinary team approach to the management of individuals undergoing RDN is recommended [18–20]. In specialized Japanese centers validated by academic societies, the Hypertension Renal Denervation Treatment (HRT) team should consist of specialists in hypertension, cardiovascular intervention, and cardiology, along with nurses, pharmacologists, and nutritionists. This team should review lifestyle modifications, medication status and drug adherence. Out-of-office BP monitoring is required to exclude white-coat hypertension, and potential causes of secondary hypertension also need to be excluded. It is important to ensure that the patient’s renal artery anatomy is suitable for RDN. Also, a shared decision-making process between the HRT team and patients is important (Fig. 2).

Data from Germany suggest that around one-third of individuals with hypertension would choose RDN over lifelong antihypertensive drug therapy [21]. In Japan, 32% of the 2392 patients with hypertension surveyed said that they had a preference for RDN, compared to antihypertensive drug therapy. The preference rates were higher in males versus females, in younger versus older patients, in those with higher rather than lower blood pressure, in patients who were less adherent versus more adherent to antihypertensive drug therapy, and in those who did versus did not have antihypertensive drug-related side effects [22]. These data highlight the importance of taking patient preference into account when determining an indication for RDN.

According to the JSH 2019 hypertension guidelines [23], the following factors need to be carefully considered both before and after RDN:

1. *Lifestyle*: Check whether adequate lifestyle modifications such as salt reduction, weight loss, smoking cessation, appropriate exercise, adequate fluid intake, good sleep, and stress management are being implemented.
2. *Medication adherence status*: If an individual is found to have poor adherence to antihypertensive drug therapy, attention should be paid to whether a partnership with the patient is being established, and appropriate action should be taken based on the hypertension treatment guidelines and targeted to the cause(s) of nonadherence. Given that polypharmacy reduces medication adherence rates [24], consider switching to a higher dose of a single agent or (preferably) a single pill combination containing multiple antihypertensive agents with different mechanisms of action [25, 26].
3. *Prescription of antihypertensive medications*: Similar to the above point, antihypertensive drug therapy should be critically evaluated, with consideration given to titrating the dosage of current antihypertensive agents or adding antihypertensives that have a complementary mechanism of action. However, it is important to note that increasing the number of antihypertensive agents does not necessarily improve the rate of BP control [27].
4. *Correct BP measurement, and target BP*: Ensure that BP measurements, including out-of-office BP, are being taken correctly and consistently, as specified by current recommendations [15, 23, 28–34], and that the chosen target BP is appropriate.
5. *Exclude causes of secondary hypertension*: Careful examination should be performed to exclude the presence of secondary hypertension, including drug-induced hypertension (e.g., associated with the use of glycyrrhizinic acid, non-steroidal anti-inflammatory drugs, and health foods). Exclusion of possible primary aldosteronism is essential to identify individuals who will respond to RDN [10].

### Indication

Transcatheter RDN is an effective BP-lowering treatment for resistant hypertension and “conditioned” uncontrolled hypertension despite appropriate treatment such as lifestyle modification and antihypertensive drug therapy (Graphical abstract).

After exclusion of the white-coat effect and secondary hypertension, especially primary aldosteronism, resistant or uncontrolled hypertension is defined as follows; office BP (≥ 140/90 mmHg) and/or out-of-office BP (24 h ambulatory BP ≥ 130/80 mmHg, daytime ambulatory BP ≥ 135/85 mmHg, nighttime ambulatory BP ≥ 120/70 mmHg, morning/evening home BP ≥ 135/85 mmHg, or nighttime home BP ≥ 120/70 mmHg) (Table 1) despite adequate lifestyle modification and treatment with maximum tolerated dosages of three or more antihypertensive agents from different classes, including a diuretic (except where there is a contraindication for use of diuretics) [23].

**Table 1:** Abnormal thresholds office and out-of-office blood pressure

Blood pressure metric	Threshold*
Office BP	SBP ≥ 140 mmHg or DBP ≥ 90 mmHg
Out-of-office BP	
Ambulatory BP	
24 h	SBP ≥ 130 mmHg or DBP ≥ 80 mmHg
Daytime	SBP ≥ 135 mmHg or DBP ≥ 85 mmHg
Nighttime	SBP ≥ 120 mmHg or DBP ≥ 70 mmHg
Home BP	
Morning	SBP ≥ 135 mmHg or DBP ≥ 85 mmHg
Evening	SBP ≥ 135 mmHg or DBP ≥ 85 mmHg
Nighttime	SBP ≥ 120 mmHg or DBP ≥ 70 mmHg

*Indication for renal denervation is office blood pressure above the defined threshold plus at least one out-of-office BP measurement above the defined thresholds

*BP* blood pressure, *DBP* diastolic blood pressure, *SBP* systolic blood pressure

“Conditioned” uncontrolled hypertension is defined as inability to up-titrate antihypertensive medication due to side effects, the presence of complications, or reduced quality of life despite adequate lifestyle modifications. This includes patients who are intolerant of antihypertensive drugs (i.e., calcium channel blockers, angiotensin converting enzyme inhibitors, angiotensin receptor blockers, thiazide-like diuretics, mineralocorticoid receptor blockers, and beta- or alpha-adrenergic blockers), or those with orthostatic hypertension [35, 36], morning hypertension [16, 37–41], nocturnal hypertension [16, 37–41], or sleep apnea (unable to use continuous positive airway pressure) [16, 37, 42, 43], atrial fibrillation [2, 44, 45], ventricular arrythmia [2, 46], or heart failure [2, 47]. Individuals with any of these conditions are at high cardiovascular risk due to increased sympathetic activity. However, at the present, the level of evidence for RDN in patients with these conditions is low because there is an absence of data from randomized controlled trials with a sham control group.

Individuals with renal aneurysm, renal artery stenosis or unsuitable renal artery anatomy (based on contrast-enhanced computed tomography evaluation), and those with an estimated glomerular filtration rate of < 30 ml/min/1.73m^2^ should not undergo RDN. In Japan, it has been estimated that a substantial proportion of individuals with hypertension would potentially be eligible for RDN [48].

### Evidence for BP-lowering effects

The design and findings of seven sham-controlled clinical trials of RDN that had a sample size of > 100 patients are summarized in Table 2 [6–12]. All of the trials used ambulatory BP-monitoring metrics as the primary endpoint, apart from SYMPLICITY HTN-3 which used office SBP. The three OFF MED trials showed that RDN significantly reduced ambulatory BP compared with the sham control [7, 8, 11], indicating that RDN significantly lowered BP throughout the 24 h period. In the ON MED trials, RDN significantly reduced daytime or 24 h ambulatory BP from baseline (by 6.5 to 8.0 mmHg) [9, 10, 12]. However, in two of these trials (REQUIRE and SPYRAL HTN-ON MED expansion [10, 12]) there was also a significant reduction from baseline in ambulatory BP in the sham control group, meaning that there was no significant between-group difference in the BP reduction. In contrast, the RADIANCE TRIO study documented a significant difference in daytime BP reduction from baseline between the RDN and sham control groups [9].

**Table 2:** Summary of seven sham-controlled, randomized trials of radiofrequency or ultrasound renal denervation that had a sample size of ≥ 100 patients

Author, date	Study	Device	Population	Renal function, eGFR in ml/min/1.73m^2^	Renal artery site treated	N (RDN/sham)	Primary endpoint	BP reduction, mmHg	Between-group p-value
Bhatt et al. [6]	SYMPLICITY HTN-3	SYMPLICITY FLEX	Resistant HTN (≥ 3 meds)	≥ 45	main	364/171	Office SBP at 6 months	RDN: 14.1 Sham: 11.7	0.26
Azizi et al. [7]	RADIANCE-HTN SOLO	PARADISE	Untreated HTN	≥ 40	main	74/72	Daytime SBP at 2 months	RDN: 8.5 Sham: 2.2	< 0.001
Bohm et al. [8]	SPYRAL HTN-OFF MED pivotal	SYMPLICITY SPYRAL	Untreated HTN	≥ 45	main + branch	166/165	24 h SBP at 3 months	RDN: 4.7 Sham: 0.6	< 0.001
Azizi et al. [9]	RADIANCE-HTN TRIO	PARADISE	Resistant HTN (≥ 3 meds)	≥ 40	main	69/67	Daytime SBP at 2 months	RDN: 8.0 Sham: 3.0	0.022
Kario et al. [10]	REQUIRE	PARADISE	Resistant HTN (≥ 3 meds)	≥ 40	main	69/67	24 h SBP at 3 months	RDN: 6.6 Sham: 6.5	0.971
Azizi et al. [11]	RADIANCE-II	PARADISE	Untreated HTN	≥ 40	main	150/74	Daytime SBP at 2 months	RDN: 7.9 Sham: 1.8	< 0.00001
Kandzari et al. [12]	SPYRAL HTN-ON MED expansion	SYMPLICITY SPYRAL	Resistant/uncontrolled HTN (1–3 meds)	≥ 45	main + branch	206/131	24 h SBP at 6 months	RDN: 6.5 Sham: 4.5	0.12

*BP* blood pressure, *HTN* hypertension; *meds* antihypertensive medications, *RDN* renal denervation, *SBP* systolic blood pressure

An additional analysis of REQUIRE, exhibited no significant inter-group difference in the whole patients [10], showed that the sham group had poor adherence to antihypertensive drug therapy at baseline, which improved after the RDN procedure [49]. This likely contributed to the marked post-procedure reduction in BP in the sham group. A similar pharmacologic dilution of the RDN treatment effect was seen in the SPYRAL HTN-ON MED expansion, where there was also an unexpectedly large reduction in BP in the sham group [12]. The number of antihypertensives being used at 3 and 6 months after RDN was greater in the sham versus RDN group. In contrast, minimal difference in the changes in antihypertensive medication between treatment groups during follow-up meant that RDN was significantly more effective than sham control with respect to reductions in office and 24 h BP in the SPYRAL HTN-ON MED trial [50].

All currently published data, with the exception of the REQUIRE study, found a significant difference between RDN and sham control with respect to reductions in nighttime BP [7–9, 11, 12, 37], which is an important target for cardiovascular risk reduction [51–56]. Finally, based on currently available data, there does not seem to be any relevant differences in the magnitude of BP-lowering effects after radiofrequency RDN and ultrasound RDN [57].

### Place in hypertension management

It seems reasonable that RDN can be used in combination with antihypertensive drug therapy for the treatment of “true” uncontrolled or resistant hypertension with high office and out-of-office BP because achieving control of nocturnal and morning hypertension is difficult using drug treatment alone. Most current antihypertensive agents, even those with a longer half-life, have a limited 24 h BP-lowering effect. The HI-JAMP study, which used the same “all-in-one” BP-monitoring device to measure office, home and ambulatory BPs, found that about one-third of individuals had uncontrolled office and daytime ambulatory BP, and rates of uncontrolled morning hypertension and nocturnal hypertension were around 45% and 55%, respectively, even in those being treated with two or more antihypertensive agents [27]. This is relevant because uncontrolled nocturnal and morning hypertension, and a riser pattern of nocturnal BP are associated with increased risk for cardiovascular events, including heart failure [15, 23, 51–53, 58, 59]. Therefore, it seems reasonable to infer that the long-term “always on”, 24 h BP-lowering effect achieved after RDN could contribute to a reduction in cardiovascular disease events [41, 54, 60].

### Japan Renal Denervation (J-RED) Registry

All individuals who undergo RDN in Japan have to be registered in the J-RED registry and have follow-up of office, ambulatory, and home BPs for 2 years. This all-case registration study will provide us the real-world data on the BP-lowering effects and its characteristics by RDN. Besides, the optional 10-year follow-up after RDN may give an insight to the impact of RDN on cardiovascular outcomes.(Table 3). These longer-term follow-up data can then be compared with historical cohorts evaluated using ambulatory or home BP monitoring, such as those enrolled in the J-HOP, JAMP, and HI-JAMP studies [27, 51, 52, 58, 61]. This should allow evaluation of the clinical benefit of RDN compared with antihypertensive drug therapy only. In addition, along with global registries [62, 63], J-RED will provide important data in Japanese individuals, allowing any potential ethnic differences in the effects of RDN on BP and cardiovascular outcomes to be determined.

**Table 3:** Follow-up assessments in the Japan REnal Denervation (J-RED) registry

	Before RDN	At RDN	Just after RDN	Mandatory 2-year follow-up after RDN				Optional 10-year follow-up after RDN							
				3 mo	6 mo	12 mo	24 mo	3 yr	4 yr	5 yr	6 yr	7 yr	8 yr	9 yr	10 yr
Patient characteristics (disease history, height, etc.)	x														
Body weight	x		x	x	x	x	x	x	x	x	x	x	x	x	x
RDN procedure		x													
Office BP (trough)	x		x	x	x	x	x	x	x	x	x	x	x	x	x
24 h ABPM	x			x	x	x	x	x	x	x	x	x	x	x	x
Home BP measurement (5 days)	x			x	x	x	x	x	x	x	x	x	x	x	x
Blood tests	x		x	x	x	x	x	x	x	x	x	x	x	x	x
Creatinine	x		x	x	x	x	x	x	x	x	x	x	x	x	x
NT-proBNP	x		x	x	x	x	x	x	x	x	x	x	x	x	x
HbA1c	x		x	x	x	x	x	x	x	x	x	x	x	x	x
LDL cholesterol	x		x	x	x	x	x	x	x	x	x	x	x	x	x
Proteinuria	x		x	x	x	x	x	x	x	x	x	x	x	x	x
ECG, LVH, SV1 + RV5 mm	x		x	x	x	x	x	x	x	x	x	x	x	x	x
Atrial fibrillation	x		x	x	x	x	x	x	x	x	x	x	x	x	x
Renal artery assessment															
Angiography		x													
Computed tomography	x				x										
Clinical outcomes (ASCVD, HF, diabetes, CKD, AF, hypotension, CV death, all-cause death) & complications				x	x	x	x	x	x	x	x	x	x	x	x
Antihypertensives (number of classes & dose)	x			x	x	x	x	x	x	x	x	x	x	x	x

*ABPM* ambulatory blood pressure monitoring, *AF* atrial fibrillation, *ASCVD* atherosclerotic cardiovascular disease, *BP* blood pressure, *CKD* chronic kidney disease, *CV* cardiovascular, *ECG* electrocardiogram, *HbA1c* glycosylated haemoglobin, *HF* heart failure, *LDL* low-density lipoprotein, *LVH* left ventricular hypertrophy, *mo* months, *NT-proBNP* N-terminal pro B-type natriuretic peptide, *RDN* renal denervation, *SV1 + RV5* electrocardiogram criteria used in the diagnosis of left ventricular hypertrophy, *yr* years

The Joint RDN Committee of the JSH/CVIT/JCS from Japan will also ensure that the J-RED registry captures important safety data relating to the RDN procedure, and will make sure that facilities enroll all individuals undergoing RDN into the registry. The aim is to have RDN performed at appropriately qualified, high-quality facilities. In addition, it is important to provide continuing education for all members of multidisciplinary treatment teams.

## Conclusion and perspectives

This Japanese consensus statement has a strong focus on the effectiveness and safety of RDN. RDN effectively reduces BP throughout the 24 h period. The BP-lowering effect of RDN is not impacted by adherence and overcomes several limitations of antihypertensive drug therapy, such as effective, long-term control of early morning and nocturnal BP. Knowledge about the impact of RDN on hard cardiovascular clinical outcomes, such as rates of stroke, myocardial infarction, heart failure and aortic dissection, will grow as clinical evidence accumulates. In addition, future advances in technology may improve the effectiveness of RDN. Therefore, Joint RDN Committee will contribute to reviewing the indications for RDN and facility accreditation annually, and update information on guidance as necessary to ensure optimum use of the RDN procedure to reduce BP in patients with hypertension.

## Data Availability

Not applicable.
